# The Leucocyte Telomere Length, *GSTM1* and *GSTT1* Null Genotypes and the Risk of Chronic Obstructive Pulmonary Disease

**DOI:** 10.3390/cimb44080257

**Published:** 2022-08-20

**Authors:** Tanya Tacheva, Shanbeh Zienolddiny-Narui, Dimo Dimov, Denitsa Vlaykova, Iva Miteva, Tatyana Vlaykova

**Affiliations:** 1 Department of Medical Chemistry and Biochemistry, Medical Faculty, Trakia University, 6000 Stara Zagora, Bulgaria; 2 Section for Toxicology and Biological Work Environment, National Institute of Occupational Health, NO-036 Oslo, Norway; 3 Department of Occupational Medicine, Faculty of Public Health, Medical University-Sofia, 1431 Sofia, Bulgaria; 4 Department of Medical Biochemistry, Medical University-Plovdiv, 4002 Plovdiv, Bulgaria

**Keywords:** COPD, GST, polymorphisms, telomeres

## Abstract

**Simple Summary:**

Chronic obstructive pulmonary disease (COPD) is characterized by chronic inflammation and oxidative stress, both in the airways and blood, and in other organs. Elevated oxidative stress and inflammation have been reported to affect leucocyte telomere length (LTL). We explored the link between *GSTM1* and *GSTT1* gene polymorphisms, LTL and COPD risk. For *GSTM1* and *GSTT1*, we genotyped 152 COPD patients and 131 non-affected controls, while for TL, we assessed 91 patients and 88 controls. There was a significant difference in *GSTM1* null genotype frequency between the patients and controls (0.59 vs. 0.38, *p* ≤ 0.000), but such was not found for *GSTT1* (*p* = 0.192). COPD patients carrying the *GSTM1* null genotype had shorter telomeres compared to those carrying the non-null genotype (15,720 bp vs. 22,442 bp, *p* = 0.008); and in controls, the opposite occurred (31,354 bp vs. 17,800 bp, *p* = 0.020). According to our results GSTM1, but not GSTT1, null genotypes might play role in leucocyte telomere shortening, and thus be involved in the pathogenesis of COPD.

**Abstract:**

Chronic obstructive pulmonary disease (COPD) is characterized by chronic inflammation and oxidative stress both in the airways and blood and other organs. Elevated oxidative stress and inflammation have been reported to affect leucocyte telomere length (LTL). Glutathione S-transferase (GST) enzymes are a large family of xenobiotic-metabolizing enzymes that utilize different ROS products. We aimed to explore the link between *GSTM1* and *GSTT1* gene polymorphisms, LTL and COPD risk. For *GSTM1*, we genotyped 152 COPD patients and 131 non-affected controls; for *GSTT1*, we genotyped 149 COPD patients and 130 controls. We were able to assess TL for 91 patients and 88 controls. There was a significant difference in the *GSTM1* null genotype frequency between the patients and controls (0.59 vs. 0.38, *p* ≤ 0.000), but such was not found for *GSTT1* (*p* = 0.192). When combining both polymorphisms, we obtained a significantly greater presence of at least one null genotype among patients (0.12 vs. 0.05, *p* = 0.027). An association between *GSTT1* and LTL was not found. COPD patients carrying the *GSTM1* null genotype had shorter telomeres compared to those carrying the non-null genotype (15,720 bp vs. 22,442 bp, *p* = 0.008); as for the controls, it was the opposite (31,354 bp vs. 17,800 bp, *p* = 0.020). The significance in both groups remained when combining *GSTM1* and *GSTT1* (COPD (at least one null) 16,409 bp vs. COPD (non-null) 22,092 bp, *p* = 0.029; control (at least one null) 29,666 bp vs. control (non-null) 16,370 bp, *p* = 0.027). The total glutathione level in *GSTM1* non-null controls was higher compared to the null genotype (15.39 ng/mL vs. 5.53 ng/mL, *p* = 0.002). In COPD patients, we found no association (*p* = 0.301). In conclusion, according to our results, *GSTM1*, but not *GSTT*1, null genotypes might play a role in leucocyte telomere shortening, and thus be involved in the pathogenesis of COPD.

## 1. Introduction

Chronic obstructive pulmonary disease (COPD) is one of the most common diseases of the airways, and possesses a large number of phenotypes [1]. It is characterized by irreversible airflow obstruction due to fibrosis of the small airways, and mucus secretion and air trapping as a result of emphysema. Many risk factors play a role in the pathogenesis of this disease, but the most common are smoking and environmental and indoor pollution (e.g., biomass exposure) [2]. The inhalation of tobacco smoke and other exogenous particles result in the formation of ROS directly or indirectly. Tobacco smoke is rich in ROS, as both the gas and tar phases contain large amounts of free radicals. ROS in the gas phase are generated during the combustion of tobacco, and are inhaled by the smoker as part of the mainstream smoke. These oxidants lead to the production of secondary ROS by inflammatory and epithelial cells within the lung as part of an inflammatory immune response [3].

Local and systemic inflammation and oxidative stress are related to the pathophysiological changes in COPD. Inflammation occurs usually as a response to pathogens (bacteria, viruses) and/or inhalation of cigarette smoke and other noxious particles [4]. The oxidative stress associated with COPD is a result of either endogenous production of reactive oxygen species (ROS) by inflammatory cells, or due to the direct oxidative damage by the cigarette smoke [5,6]. Oxidative stress has a wide range of effects, one of which is telomere length shortening [7,8].

Telomeres are nucleoprotein structures at the end of the chromosomes that consist of the following hexameric tandem repeat: TTAGGG. Due to the replication end problem, the telomeres are shortened in each cell division. It has been found that when telomeres are shortened beyond a critical level (under 6000–8000 base pairs), the cell loses its ability to divide and becomes senescent or undergoes apoptosis [9,10]. In some cells, such as stem cells, an enzyme known as telomerase maintains the telomeres length (TL). The TL varies widely between individuals, and even between different cells in one organism, as there are variety of factors (endogenous and exogenous) that impact TL [11]. It has been found that leukocyte telomere length (LTL) decreases with age, and is associated with increased risk of age-related diseases, such as cardiovascular diseases, diabetes, rheumatoid arthritis, and COPD [12,13,14].

Inflammation and oxidative stress have been found to reflect on telomere length [15]. In an in vitro study, it has been shown that in endothelial cells, ROS decreases telomerase activity and the level of nuclear human telomerase reverse transcriptase (hTERT) [16]. On the other hand, reductions in oxidative stress and antioxidants have been shown to decrease the rate of telomere shortening and delay the beginning of senescence via mechanisms related to telomerase activity [11,16,17,18].

Keeping in mind that oxidative stress is a major factor in COPD pathogenesis and telomere shortening, it could be suggested that antioxidants may have plausible effects in the prevention of both. Indeed, antioxidants have been shown to improve the symptoms in COPD and delay ageing [5,19]. As one of the most important antioxidants in the body, we hypothesize that glutathione might reflect the LTL.

Glutathione S-transferases (GSTs) are a superfamily of enzymes that catalyze the conjugation of reduced glutathione (GSH) to a wide variety of exogenous and endogenous toxic compounds with electrophilic sites, including cigarette smoke and ROS, and protect DNA from oxidative damage. So far, GSTs have been classified into eight classes: α (GSTA), μ (GSTM), θ (GSTT), π (GSTP), σ (GSTS), κ (GSTK), ο (GSTO) and τ (GSTZ) [20]. It has been found that most of the genes encoding GST isoenzymes are polymorphic. A greater degree of polymorphism has been reported for *GSTM1* and *GSTT1* genes as homozygous deletion results in the lack of the protein product and loss of enzymatic activity [21,22].

There is evidence that shorter telomeres and null variants of *GSTM1* and *GSTT1* are associated with increased risk of age-related diseases, including COPD [13,23,24].

In this study, we aimed to explore the link between the *GSTM1* and *GSTT1* null genotypes, total glutathione, LTL and the risk of COPD.

## 2. Methods and Materials

### 2.1. Patients and Controls

In our study were included 152 patients with COPD and 131 control individuals that were non-affected by any lung diseases. All patients and controls were from the region of Stara Zagora, Bulgaria. The recruitment of the patients was conducted in the Clinic of Internal Medicine, University Hospital, Trakia University, Stara Zagora, Bulgaria. All inclusion criteria for COPD were described in our previous study [25].

The available demographic and clinical data of the examined groups are presented in Table 1.

The protocol of the current study (number 10/05.06.2019) was issued by the ethics committee at the Medical Faculty of Trakia University, Stara Zagora, Bulgaria. Written informed consent was obtained from each of the participants before the study.

### 2.2. Isolation of DNA

The isolation of genomic DNA was performed using a commercial kit (GenElute™ mammalian genomic DNA miniprep kit, Sigma- G1N70, Merck KGaA, Darmstadt, Germany). As described earlier, the purity of the isolated DNA was assessed spectrophotometrically [25].

### 2.3. Genotyping

The null polymorphisms in *GSTM1* and *GSTT1* were assessed using multiplex (duplex) PCR [26], which we modified, as described in detail in a previous study [27]. *GSTP1* was chosen as a reference gene for assessing the amplification process and presence of sufficient amount of DNA template. The primers and the size of the amplification products, as well as the temperature profile of the PCR reactions, are provided in Table 2.

As part of assessing the purity of the PCR reaction, positive and negative (blank) controls were included in all PCRs. We used, as positive controls, samples that in previous analyses were genotyped as *GSTM1* non-null and *GSTT1* non-null [27]. The negative controls were reactions performed without total DNA as a template. PCR products were analyzed on 2.5% agarose gel, stained with ethidium bromide, and documented with a gel documentation system (Syngene, Synoptics Ltd., Cambridge, UK).

### 2.4. Leukocyte Telomere Length (LTL) Measurement

The telomere length was measured using SYBR green qPCR method, described in detail previously [25].

### 2.5. Serum Total Glutathione Measurement

The total glutathione (GSSG + GSH) concentration was evaluated in serum deproteinized with 5% 5-sulfosalicylic acid. The applied method is a kinetic assay (Sigma, glutathione assay kit; CS0260) that measures the reduction in 5,5-dithiobis-(2-nitrobenzoic) acid (DTNB) by GSH to TNB, which is detected spectrophotometrically at 412 nm.

### 2.6. Statistical Analyses

Statistical analyses were performed using SPSS 16.0 for Windows (IBM, Chicago, IL). After assessing the normality of the distribution of the continuous variables, they were compared either by a Student’s t-test or one-way ANOVA with LSD post hoc analysis, or using a Mann–Whitney U test or Kruskal–Wallis H test, for those with normal and non-normal distributions, respectively. The correlations between the continuous variables were assessed using a Pearson’s correlation test.

The differences in genotype distribution between the patient and control groups were assessed with χ^2^ tests in 2 × 2 contingency tables. The odds ratios (OR) and 95% confidence intervals (CI) were obtained by applying the binary logistic regression. Outputs with *p* < 0.05 were considered statistically significant.

## 3. Results

**Figure 1:**
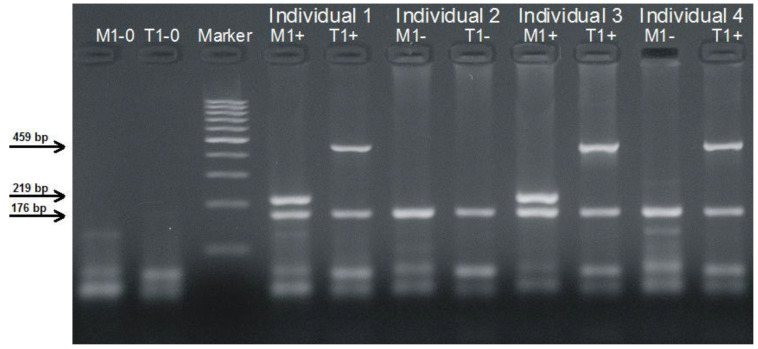
Genotyping of *GSTM1* and *GSTT1* by duplex PCRs. Non-null *GSTM1* genotype individual 1 (M1+) and 3 (M1+); null *GSTM1* genotype individual 2 (M1–) and 4 (M1–); non-null *GSTT1* genotype individual 1 (T1+), 3 (T1+) and 4 (T1+); null *GSTT1* genotype individual 2 (T1–); lines 1 and 2 (M1-0 and T1-0) are negative controls for *GSTM1* and *GSTT1* duplex PCRs; line 3 (marker) is a 100-bp DNA ladder (Thermo Fisher Scientific Inc, Waltham, MA, USA).

The *GSTM1* and *GSTT1* null polymorphisms were analyzed in multiplex (duplex) PCR reactions, using *GSTP1* as a reference gene in each of the reactions. In the electrophoresis gel, one individual that was homozygous for the *GSTM1* null polymorphism (*GSTM1* null) had only one visible band of 176 bp of the PCR reaction products corresponding to the PCR product of the reference *GSTP1* gene, while another person that carried one or two wildtype alleles of *GSTM1* (*GSTM1* non-null) had two bands: a 219-bp band of the *GSTM1* PCR product and a 176-bp band of the *GSTP1* reference gene (Figure 1). Analogously, an individual with at least one wildtype *GSTT1* allele (*GSTT1* non-null) had, in the electrophoretic gel, two PCR products: a band of 459 bp for *GSTT1*, and a band of 176 bp for the *GSTP1* reference gene. In contrast, a person homozygous for the *GSTT1* null polymorphism (*GSTT1* null) had, in the electrophoretic gel, only one PCR product of 176 bp, corresponding to the *GSTP1* reference gene.

The *GSTM1* null genotypes were found to be more frequent in the COPD patient group, implying a 2.35-fold higher risk of COPD (*p* ≤ 0.000, Table 3). For *GSTT1*, there was no significant difference of genotype distributions between patients and controls (*p* = 0.192, Table 3).

When we analyzed the combined effect of *GSTM1* and *GSTT1* null polymorphisms, we found a significant prevalence of at least one null genotype (either *GSTM1* or *GSTT1* or both null genotypes simultaneously) in COPD patients compared to controls (*p* = 0.027, Table 3). Thus, individuals that carry at least one null variant have a 2.84-fold higher risk of developing COPD. The above associations of the effects as risk factors of *GSTM1* null variant, or the presence of at least one null variant, remained significant after adjustment for sex and age (Table 3).

**Figure 2:**
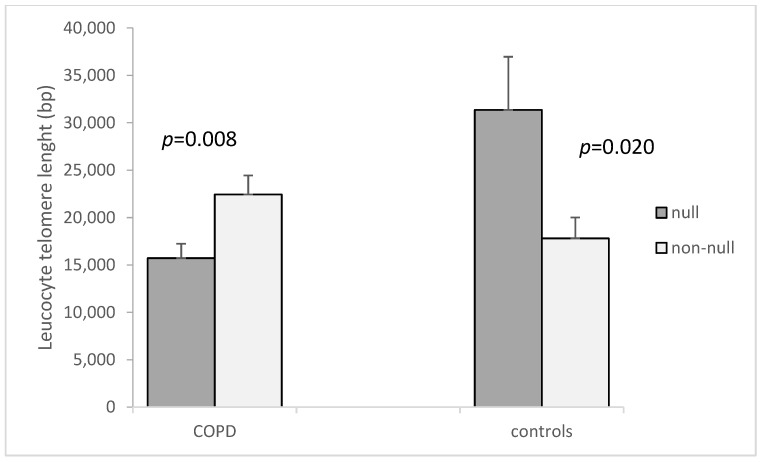
Leucocyte telomere length in COPD patients and controls according to presence of *GSTM1* null variant. The LTL is presented as mean ± standard error of mean (SEM).

When analyzing the possible relationship between the LTL and the carrying of *GSTM1* or *GSTT1* null variants, we found that patients that carry the *GSTM1* null variant had shorter telomeres in circulating leucocytes compared to those carrying the non-null genotype (15,720 ± 1514.6 bp vs. 22,442 ± 1995.9 bp, *p* = 0.008, Figure 2). Interestingly, in the control group, those carrying the *GSTM1* non-null genotype had shorter telomeres compared to those with the null genotype (17,800 ± 2217.3 bp vs. 31,354 ± 5608.8 bp, *p* = 0.020, Figure 2). Although not significant, controls with the null *GSTM1* variant were younger (57.40 ± 1.71 (SEM) years) than those with the non-null *GSTM1* genotype (60.54 ± 1.31 (SEM) years, *p* = 0.174). In the patient group, mean age was comparable for the *GSTM1* null variant and non-null variant carriers (66.51 ± 0.95 (SEM) years vs. 66.89 ± 1.29 (SEM) years, *p* = 0.777).

No association between LTL and *GSTT1* was found, either in patients (non-null: 18,588 ± 1368.07 bp vs. null: 18,097 ± 3638.6 bp, *p* = 0.901), or in controls (non-null: 22,511 ± 2854.6 bp vs. null: 29,745 ± 9498 bp, *p* = 0.328).

**Figure 3:**
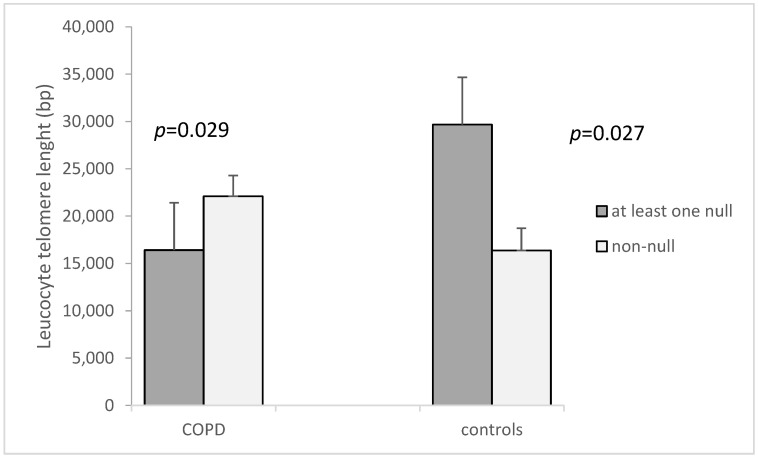
Leucocyte telomere length in COPD patients and controls in *GSTM1* and *GSTT1* combined genotypes (carriers of either *GSTM1* and *GSTT1* non-null genotype or at least one null genotype). The LTL is presented as mean ± standard error of mean (SEM).

A similar association as that found for the *GSTM1* genotype and LTL was found when combining both *GSTM1* and *GSTT1*. COPD patient carriers of at least one null variant had shorter telomeres (16,409 ± 1478.4 bp vs. 22,092 ± 2190 bp, *p* = 0.029, Figure 3). In controls, this was the opposite; individuals with the non-null genotype had shorter LTLs (16,370 ± 2358.6 bp vs. 29,666 ± 4664.2 bp, *p* = 0.027, Figure 3).

**Figure 4:**
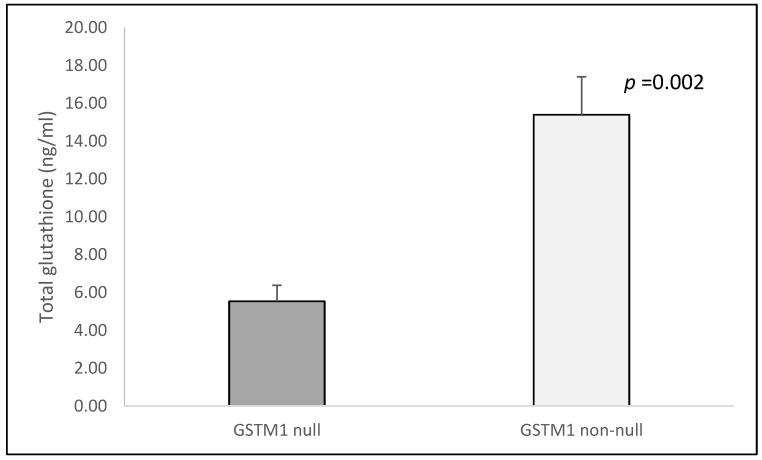
Total glutathione levels in controls according to *GSTM1* genotypes. The total glutathione is presented as mean ± standard error of mean (SEM).

The serum levels of total glutathione were significantly higher in controls with the *GSTM1* non-null genotype in comparison to the carriers of the null *GSTM1* genotype (15.39 ± 2.01 ng/mL vs. 5.53 ± 0.85 ng/mL, *p* = 0.002, Figure 4). In COPD patients, we found no difference (non-null: 9.53 ± 2.27 ng/mL vs. null: 13.39 ± 2.26 ng/mL, *p* = 0.301).

No association was found for *GSTT1* and total glutathione, both in patients (null: 7.29 ± 1.54 ng/mL vs. non-null: 13.43 ± 2.07 ng/mL, *p* = 0.205), and controls (null: 10.91 ± 4.15 ng/mL vs. non-null: 11.58 ± 2.06 ng/mL, *p* = 0.885).

**Figure 5:**
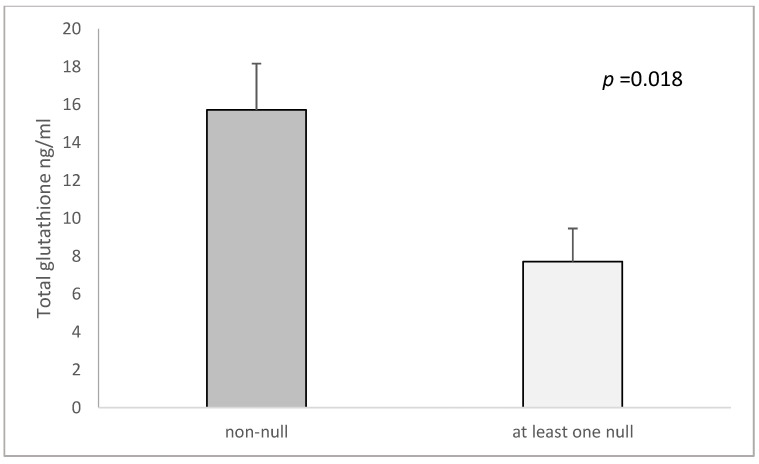
Total glutathione levels in controls in *GSTM1* and *GSTT1* combined genotypes (carriers of either GSTM1 and GSTT1 non-null genotype or at least one null genotype). The total glutathione is presented as mean ± standard error of mean (SEM).

Controls with the non-null genotype of both *GSTM1* and *GSTT1* had higher total glutathione in serum in contrast with those carrying at least one null variant (15.72 ± 2.44 ng/mL vs. 7.71 ± 1.75 ng/mL, *p* = 0.018, Figure 5). In COPD patients, there was a lack of difference in the serum levels of total glutathione between the genotypes (non-null: 9.62 ± 2.59 ng/mL vs. at least one null: 13.15 ± 2.15 ng/mL, *p* = 0.365).

We failed to find a correlation between serum total glutathione and LTL in COPD patients (r = −0.005, *p* = 0.980) and controls (r = −0.273, *p* = 0.446).

The concentration of total glutathione in the serum (ng/mL) and the LTL (bp) in controls and COPD patients are presented in Table 4.

## 4. Discussion

It is known that one of the factors involved in the pathogenesis of COPD is oxidative stress, as it might be the result of either the overproduction of ROS or impaired antioxidant defense systems (or both). One of the major triggers of ROS production and inflammation in COPD are cigarette smoking and inhalation of environmental and occupational pollutants (e.g., wood smoke, coal dust, etc.) [28].

Following inhalation of exogenous particles, inflammatory cells, including macrophages and neutrophils, accumulate in the lungs, generating a large amount of endogenous ROS in order to protect the lungs against exogenous pollutants via phagocytosis. However, in chronic inflammatory conditions, such as COPD, macrophages and neutrophils become a persistent source of oxidants, leading to tissue injury and further oxidative stress [29,30].

GSTs are enzymes that catalyze the conjugation of GSH with a variety of toxic compounds, including oxidative intermediates and ROS, and it has been found that they are expressed in the airway epithelium and alveolar macrophages [5,6,31,32]. Most of the genes encoding the GSTs are polymorphic, as the null variants, due to the homozygous deletions of the *GSTM1* and *GSTT1* genes, might result in the lack of GST-mu and GST-theta enzyme activity [21].

In our study, we found higher prevalence of the *GSTM1* null variant and at least one null variant of either *GSTM1* or *GSTT1* in COPD patients. Based on the experimental studies of Seidegård et al. [33], as well as the results of Lee et al. [34], concerning the effects of the null variant of *GSTM1* and *GSTT1* genes on enzyme activity and enzyme protein levels, we suggest that the loss of enzymatic activity in COPD patients that carry the null *GSTM1* variant results in altered antioxidant defense and the detoxification of ROS and their products. This additionally contributes to the pathological changes present in COPD due to oxidation and inflammation

As mentioned above, in our study, we found that the *GSTM1* null variant, as well as the presence of at least one null *GSTM1* or *GSTT1* variants, determines a more than twofold higher risk for developing COPD. However, the results published in the scientific literature on the association of *GSTM1* and *GSTT1* polymorphisms with COPD are rather controversial.

In accordance with our findings, higher frequencies of the *GSTM1* null genotype in COPD patients and no significant differences between patients and controls for the *GSTT1* null polymorphism have been reported for a Serbian population [35]. Conversely, Zidzik et al. reported no association between null *GSTM1* and *GSTT1* genotypes and the risk of COPD [36]. The same has been found for Croatian males with stable COPD [18]. Studies conducted in India [37], Taiwan [38], Korea [39] and Turkey [40] have also shown controversial results, which might be associated with population differences.

We should not underestimate the fact that many other enzymes and non-enzymatic antioxidants might also impact the progress of COPD. It is well known that the substrate specificities of the different isoenzymes of GSTs overlap. In our study, we did not explore the enzyme activity of GSTs, which might have helped us to take a closer look into the multifactorial pathogenesis of the disease. The fact that there is a large difference in the results from a variety of studies show that as well as the polymorphisms in *GSTM1* and *GSTT1*, other factors such as epigenetic and posttranslational modifications might influence the function of the enzymes, and, furthermore, predisposition to the disease. In addition, we did not find any differences in the genotype distribution among patients with different severities of COPD (GOLD stages), suggesting the role of factors varying from the deletion polymorphisms of *GSTM1* and *GSTT1* in the progression of this disease.

The impaired antioxidant defense will reflect on different cellular structures [41], as telomeres are highly susceptible to oxidative stress. This notion was proven in in vitro and in vivo studies, which have reported that oxidative stress leads to the telomere shortening [42,43,44]. In this respect, it is not surprising that our COPD patients with the *GSTM1* null variant, and those with at least one null *GSTM1* or *GSTT1* variant, had significantly shorter LTLs compared to those with non-null genotypes. Except the loss of enzymatic GST activity that might be a result of the deletion polymorphisms in *GSTM1* and/or *GSTT1*, in COPD, there is increased production of ROS [5,6], which will additionally contribute to LTL shortening.

It has been proposed that short telomeres could contribute to inflammation associated with COPD by inducing senescence, which upregulates the secretion of inflammatory factors systemically [17].

Telomeres are rich in guanine, and thus, they are more sensitive to oxidative damage [44]. It has been found that a variety of environmental and behavioral factors associated with COPD (tobacco smoking, air pollution, etc.) that lead to oxidative stress are able to influence LTL [28,45,46]. Oxidation may lead to DNA damage, resulting in double- or single-strand DNA breaks that eventually lead to telomere shortening [44]. Telomere shortening triggers senescence and loss of replicative capacity, as LTL is inversely related to age, and has been found to be associated with increased risk of age-related diseases, including COPD [47,48,49]. Another finding that emphasizes the role of the oxidative stress in telomere shortening is that in women, the average telomere length is higher [50]. It is known that estrogen protects the body from ROS formation and acts as an antioxidant [51]. In our previous study, we also described such an association with the gender, and suggested that LTL might be influenced by age and sex [25].

GSTM1 and GSTT1 enzymes decrease oxidative damage and prevent induced genotoxic attacks on DNA [52]. Many studies have explored the role of *GSTM1* and *GSTT1* polymorphisms in the genotoxic damage and telomere length [53,54,55].

Interestingly, we found that the controls carrying the non-null *GSTM1* genotype had shorter telomeres. Telomere metabolism is a very dynamic process that is controlled by genetic as well as environmental determinants, and is highly specific at the individual-level. In addition to oxidative stress and inflammation, there are a number of factors that reflect the length of telomeres, such as maternal exposures during pregnancy, age of the father at conception, physical activity, diet, ethnicity, etc. [11]. Sex might be a possible explanation for this result, as it is probable that estrogen or some other features have an impact on LTL rather than polymorphisms in *GSTM1* and *GSTT1*. In addition, another explanation for this peculiar observation for longer LTL in null GSTM1 controls might be age. Although the difference is not significant, the mean age of our controls with the null *GSTM1* variant was lower than of controls with the non-null genotype.

In addition to shorter LTL, in *GSTM1* non-null controls, the concentration of total glutathione was higher. This might be a compensatory mechanism reducing the risk of age-related diseases by preventing oxidative damage. Glutathione is an important intra- and extracellular component of the antioxidant defense system found in epithelial lining fluid and plasma [56]. GSH homeostasis plays an important role in the maintenance of the integrity of the lung epithelial barrier, as the inhaled noxious particles, together with endogenous ROS, may trigger a significant local and systemic inflammatory response typical of COPD [57]. Patients with chronic inflammatory lung diseases, such as COPD, have shown increased oxidative stress and decreased levels of glutathione, enhancing the inflammatory response [30].

Probably, by preventing oxidation in the lungs and systematically, GSH reduces the inflammatory response, and thus, even with shorter leucocyte telomeres, the carriers of the *GSTM1* non-null genotype may not develop COPD.

Despite exhibiting interesting and statistically significant results, our study has some limitations. One of the limitations is that we used qPCR instead of the fluorescence in situ hybridization (FISH) method, which is considered more reliable. Other limitations are the small number of individuals with measured LTL and the predominance of females in the control group. Therefore, a larger study enrolling more COPD patients and controls is warranted in order to confirm our current observations and conclusions.

## 5. Conclusions

Finding reliable biomarkers for COPD may help in the prevention and control of disease. We should not underestimate the fact that many mediators and lifestyle factors can modulate the inflammatory response and telomere length. Furthermore, the results of the role of *GSTM1* and *GSTT1* polymorphism, together with LTL, in the development and severity of COPD are still relatively diverse. However, according to our findings, *GSTM1*, but not *GSTT1*, null genotypes might play a role in leucocyte telomere shortening, and thus be involved in the pathogenesis of COPD.

## Figures and Tables

**Table 1:** Demographic and clinical data of COPD patients and controls.

Characteristics	COPD Patients	Controls
Number	(*n* = 152)	(*n* = 131)
males	114 (75%)	61 (46.6%)
females	38 (25%)	70 (53.4%)
Age at the inclusion in the study		
mean ± SD (years)	66.6 ± 9.4	59.3 ± 11.69
median (range) (years)	67 (40–88)	60 (23–85)
Age at the diagnosis of the disease		
mean ± SD (years)	61.5 ± 9.9	
median (range) (years)	62 (34–86)	
Duration of the disease		
mean ± SD (years)	5.26 ± 5.4	
median (range) (years)	4 (0–30)	
Smoking status	(*n* = 152)	(*n* = 131)
non-smokers	45 (30.2%)	59 (60.2%)
ex-smokers	66 (44.3%)	10 (10.2%)
current smokers	38 (25.5%)	29 (29.6%)
Smoking habits (packs/year)		
mean ± SD (years)	31.6 ± 14.1	16.4 ± 10.7
median (range)	30 (5–70)	15 (5–50)
COPD stage	(*n* = 152)	
GOLD II	73 (48%)	
GOLD III	69 (45.4%)	
GOLD IV	10 (6.6%)	
FEV1 % pred.		
mean ± SD	50.47 ± 13.76	96.25 ± 11.67
FEV1/FVC%		
mean ± SD	60.81 ± 8.87	81.40 ± 7.88

**Table 2:** Primer sequences, size of the PCR products, and temperature profile used in the multiplex (duplex) PCR for genotyping for null *GSTM1* and *GSTT1* polymorphisms.

Gene	Primers	Size of the PCR Product
*GSTP1* (reference gene)	F: 5′-ACC CCA GGG CTC TAT GGG AA-3′ R: 5′-TGA GGG CAC AAG CCC CT-3′	176 bp
*GSTM1*	F: 5′-GAA CTC CCT GAA AAG CTA AAG C-3′ R: 5′-GTT GGG CTC AAA TAT ACG GTG G-3′	219 bp
*GSTT1*	F: 5′-TTC CTT ACT GGT CCT CAC ATC TC-3′ R: 5′-TCA CCG GAT CAT GGC CAG CA-3′	459 bp
Temperature profile of PCR	10 min at 94 °C-predenaturation 40 cycles of: 94 °C-1 min, 62 °C-30 s, 72 °C-1 min 7 min at 72 °C-final extension	

**Table 3:** Genotype of *GSTM1* and *GSTT1* gene polymorphism in patients with COPD and controls.

	COPD Patients		Controls		OR (95% CI), *p*-Value	OR (95% CI), *p*-Value (Adjusted for Sex and Age)
	*n*	Frequency	*n*	Frequency		
***GSTM1***	*n* = 152		*n* = 131			
non-null	62	0.408	81	0.618	1.0 (reference)	1.0 (reference)
null	90	0.592	50	0.382	2.35 (1.46–3.79), ***p* ≤ 0.000**	2.49 (1.46–4.23) ***p* = 0.001**
***GSTT1***	*n* = 149		*n* = 130			
non-null	117	0.785	110	0.846	1.0 (reference)	1.0 (reference)
null	31	0.215	20	0.154	1.46 (0.79–2.69), *p* = 0.192	1.39 (0.71–2.72) *p* = 0.337
***GSTM1* and *GSTT1***	*n* = 149		*n* = 130			
both non-null	131	0.879	124	0.954	1.0 (reference)	1.0 (reference)
at least one null	18	0.121	6	0.046	2.84 (1.12–7.17), ***p* = 0.027**	2.39 (1.40–4.08) ***p* = 0.001**

**Table 4:** Concentration of total glutathione in the serum (ng/mL) and the LTL (bp) in controls and COPD patients. Data are presented as mean ± SEM.

Genotype	Total Glutathione Mean ± SEM (ng/mL)			LTL Mean ± SEM (Base Pairs)		
	Controls	Patients	*p*-Value	Controls	Patients	*p*-Value
GSTM1 non-null ^a^	15.39 ± 2.01	9.53 ± 2.27	0.023	17,800 ± 2217.3	22,442 ± 1995.9	0.146
GSTM1 null	5.53 ± 0.85	13.39 ± 2.26	0.327	31,354 ± 5608.8	15,720 ± 1514.6	0.004
GSTT1 non-null ^b^	11.58 ± 2.06	13.43 ± 2.07	0.573	22,511 ± 2854.6	18,588 ± 1368.1	0.353
GSTT1 null	10.91 ± 4.15	7.29 ± 1.54	0.376	29,745 ± 9498	18,097 ± 3638.6	0.442
both GSTM1 and GSTT1 non-null ^c^	15.72 ± 2.44	9.62 ± 2.59	0.046	16,370 ± 2358.6	22,092 ± 2190	0.147
GSTM1 and GSTT1-at least one null	7.71 ± 1.75	13.15 ± 2.15	0.511	29,666 ± 4664.2	16,409 ± 1478.4	0.006
^a^*p*-value (GSTM1 non-null vs. null)	0.002	0.301		0.020	0.008	
^b^*p*-value (GSTT1 non-null vs. null)	0.885	0.205		0.328	0.901	
^c^*p*-value (both non-null vs. at least one null)	0.018	0.365		0.027	0.029	

## Data Availability

The date of this study is available as SPSS file when requested to the authors.
